# Relationship between Operator Skill and *In Vitro* Microleakage of Different Adhesive Systems in Class V Restorations

**DOI:** 10.5402/2011/285624

**Published:** 2010-11-29

**Authors:** Audrey Guéders, Sabine Geerts

**Affiliations:** ^1^Division of Conservative and Adhesive Dentistry, Department of Dentistry, University of Liège, 4020 Liège, Belgium; ^2^CHU-Policlinique Brull, Quai G. Kurth, P.O. Box 45, 4020 Liège, Belgium

## Abstract

*Objectives*. The aim of this study was to evaluate the influence of operator skill on microleakage in class V composite restorations. *Materials and Methods*. A total of 16 dentists and 25 dental students were enrolled, and 123 extracted teeth were allocated according to the adhesive being tested: Scotchbond Multipurpose, Adper Scotchbond 1 XT, and AdheSE. Each operator was asked to restore one tooth from each experimental group: two class V cavities were cut on each tooth and each adhesive was used on the same tooth before and after instructions for its use. After filling cavities with composite (Z100), the teeth were thermocycled. *Results*. For all of the tested adhesives, the mean microleakage score was lower for the dentists than for the students. The mean scores for the three tested adhesives were statistically similar before and after instructions for use. *Conclusion*. Our results indicate that the skill of the operator has a significant influence on microleakage.

## 1. Introduction

While there have been advances in our understanding of micromechanical and chemical adhesion to dental tissues, microleakage remains the major cause of failure in composite restorations. The microleakage is also related to the bonding quality and polymerization shrinkage of adhesives. Many different adhesive systems have recently been developed by manufacturers, employing one of the three following strategies [1, 2]: Etch and Rinse (ER) adhesion, Self-Etch (SE) adhesion, or adhesion by glass ionomer cement.

Several authors have reported statistical differences among the *in vitro *performance of various tested adhesive systems [3, 4]. Significant differences were also found when different operators used the same adhesive [5, 6]. For many years, Professor Degrange and his team have run workshops for dentists to test their most common adhesive systems as well as many others [7]. In these experiments, adhesive restorations were immediately submitted to a tensile test to obtain bond strength values. The results showed that for a particular adhesive system, differences between the operators were more important than differences among the various tested adhesives [7]; accordingly, other authors have also suggested that dental adhesion is strongly operator-dependant [6, 8–11]. Thus, the purpose of the present study was to evaluate the influence of operator skill on microleakage in class V restorations using ER and SE adhesives.

## 2. Materials and Methods

For the experiment, 123 recently extracted human third molars were selected and stored in refrigerated saline solution for a maximum of 3 months, as recommended by the International Organization for Standardization (ISO, Guidance on Testing of Adhesion to Tooth Structure. International Organization for Standardization, TR 11405,1-4, Geneva, Switzerland, 1994). The collection of extracted teeth was approved by all patients and by the appropriate Ethical Committee. 

### 2.1. Bonding Procedures

Teeth were randomly assigned into three groups according to the adhesive being tested. All adhesives were tested before and after the operators were given rigorous instruction on the correct use of the materials, according to the manufacturers' instructions. The three groups were as follows: group 1, Scotchbond Multipurpose (SBMP) (3M ESPE, Dental Products, Seefeld, Germany), an ER 3-step adhesive system; group 2, Adper Scotchbond 1 XT (SB1) (3M ESPE), an ER 2-step adhesive system; group 3, AdheSE, a SE 2-step adhesive system (ADSE) (Ivoclar Vivadent, Schaan, Liechtenstein).

A total of 41 operators were enrolled in the study: 16 dentists (expert operators, EO), 11 students enrolled in second year of a Master's course (moderately experienced operators, MEO), and 14 students enrolled in first year of a Master's course (inexperienced operators, IO). Each operator received three teeth in which two cavities had been cut earlier (one each on the lingual and buccal surfaces). All cavities were drilled before the beginning of the study by the same dentist (Audrey Guéders) and were standardized for dimension and shape: each rectangular cavity (*h* × *w* × *l* = 2 × 2 × 3 mm) was prepared using a cylindrical diamond bur (diameter = 0.9 mm) at the cemento-enamel junction and the margins were butt-jointed: half in the enamel and half in the root dentin.

The study was conducted into two parts. First, the operators were asked to restore the buccal surfaces of the three teeth consecutively, using the three tested adhesives; they were given the commercial name and the type of adhesive system (ER 3-step, ER 2-step, or SE 2-step), but no instructions for their use. The operators filled the cavities with a microhybrid composite (Z100, 3M ESPE) immediately after bonding procedures. Second, the operators were given the manufacturer's instructions for each material (adhesive systems and composite) and were then asked to restore the three lingual cavities using the same adhesive system on the lingual side that had been used on the buccal side of each tooth. In other words, the tooth that received SBMP on the buccal side (before receiving the manufacturer's instructions) also received SBMP on the lingual side (after receiving the manufacturer's instructions) (Table 1); the same was the case for the other two adhesives. As recommended by the manufacturers, lingual restorations were made with two oblique increments of the microhybrid composite immediately after bonding procedures.

After completing Part 1 of the study, a total of 1, 2, or 3 grooves were drilled into the coronal portion of the buccal surface to identify the adhesive used for each specimen (SBMP, SB1, and ADSE, resp.) and also to identify the restored side before receiving the manufacturer's instructions (Table 2). 

Photopolymerization of adhesives and composite was performed with halogen lamps: Demetron LC (Kerr Corporation, Scafati, Italy) (one lamp per operator). The light output of each lamp, which was checked with a halogen radiometer before the experiment, ranged from 330 to 350 mW cm^−2^. In the second part of the study only, operators were asked to carefully follow the recommended curing times for each material: SBMP, SB1, and ADSE for 10 seconds and each increment of Z100 was cured for 20 seconds.

Each operator was designated a code to ensure blinding of the study. The composite restorations were all finished by the same operator (Audrey Guéders) using diamond drills and were polished with disks (Hawe Neos Dental, Bioggio, Switzerland) under waterspray.

### 2.2. Preparation of Specimens for Thermocycling

The apices were fixed in an autopolymerizing resin (Paladur, Heraeus-Kulzer GmbH & Co. KG, Hanau, Germany) and the specimens were immersed in saline solution for 12 weeks (refrigerated at 5°C). They were then thermocycled for 800 cycles (5–55°C) over 22 hours. After thermocycling, the teeth were immersed in 50% silver nitrate solution for 6 hours and in 25% vitamin C solution (pH of approximately 2) for 10 minutes [12].

### 2.3. Preparation of Specimens for Microscopic Observation

**Figure 1:**
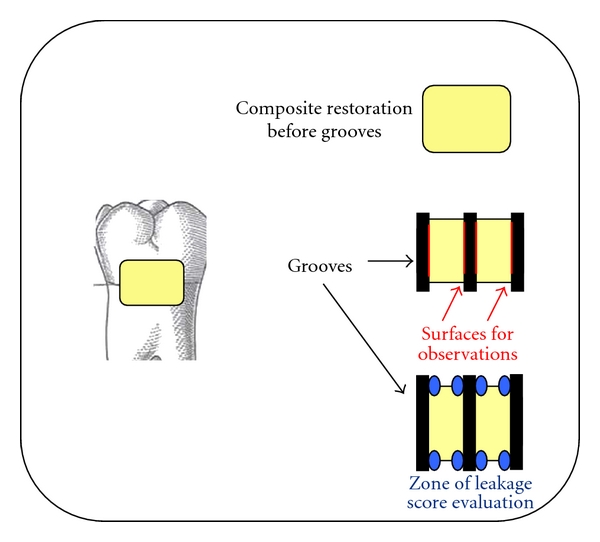
Diagram showing placement of the three grooves on each restoration, to provide eight observation areas.

After immersion, three grooves (depth, 3 mm; width, 1 mm) were drilled with diamond burs (0.9 mm diameter) in each restoration to obtain four surfaces for observation. The interfaces that occur between the tooth and the filling are described in our previous study [12]. Briefly, the cylindrical diamond drill was placed perpendicular to the restoration and three grooves were cut: one at the mesial margin, one at the distal margin, and one in the centre of the filling (Figure 1). These preparations yielded four evaluation surfaces for each restoration, for a total of 984 viewing surfaces. For each surface, one measurement of leakage was obtained in the enamel and one in the dentin (observation areas), for a total of 1968 measures.

Each section was examined at ×2 magnification using an optic microscope (Carl Zeiss, SAS, Oberkochen, Germany), and observation of each tooth was performed twice by the same operator (blinded test).

**Figure 2:**
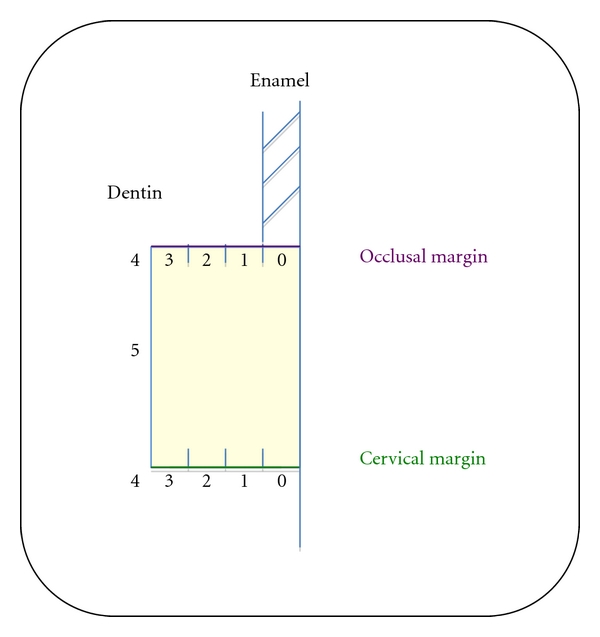
Diagram showing the 6-point evaluation scale for leakage.

The severity of leakage was evaluated using an arbitrary 6-point scale, as follows (Figure 2):

Score = 0: no leakage
Score = 1: leakage up to the enamel-dentin junction or to a depth of 0.5 mm on the radicular wall
Score = 2: leakage to a maximum of half of the lateral wall (leakage depth ≤ 1 mm)
Score = 3: leakage over half of the lateral wall (1 mm < leakage depth < 2 mm)
Score = 4: subtotal leakage over all of the lateral wall (leakage depth = 2 mm)
Score = 5: total leakage partially or entirely over the pulpal wall of the cavity (leakage depth > 2 mm).

We postulated that higher scores of microleakage (scores 3, 4, and 5) after thermocycling would indicate clinical failure of the bonding.

### 2.4. Statistical Analysis

Results are expressed as the mean ± standard deviation (±SD). Microleakage scores were analysed using generalized linear mixed models (GLMMs) assuming an ordinal logistic link function. Covariates in the model were (1) groups of operators: EO, MEO, or IO, (2) the tested adhesive systems (SBMP, SB1, or ADSE), (3) theoretical information and instructions for use, and (4) the interface (enamel or dentin). The model also accounts for repeated measurements of the various teeth. All the results were considered to be significant at the 5% critical level (*P* < .05). Statistical calculations were performed using the SAS 9.1 (version 8.2 for Windows) package.

## 3. Results

When all adhesives were considered together, statistical analysis revealed significant difference among the operator groups (*P* < .0001). Tables 3 and 4 show that EO group had a significantly lower mean score for microleakage (0.47 ± 0.59) than did the other groups (0.58 ± 0.65, *P* = .0029 for MEO; 0.67 ± 0.66, *P* < .0001 for IO). In addition, IO group showed a significantly higher mean score for microleakage compared with the MEO group (*P* = .023) (Table 4).

Tables 5 and 6 show the mean scores for microleakage according to operator skill and the tested adhesives, before and after instructions, for the three tested adhesive systems. In all operator groups, comparison of the mean scores for microleakage for the three tested adhesive systems showed no statistical difference between the periods before and after instruction.

When all groups of operators were considered together before instruction, there was no significant difference among the different adhesive systems (*P* = .42). When the analysis took into account the skill of the operator, no statistical difference was found in the EO and IO groups (*P* = .68 and *P* = .19, resp.). In contrast, significant difference was found among the three tested adhesives in the MEO group (*P* = .0019): the mean microleakage score for SBMP (0.78 ± 0.75) was greater than that for SB1 (0.48 ± 0.52; *P* = .0056) and for ADSE (0.43 ± 0.52; *P* = .001).

After the operators had been given the manufacturer's instructions, considering all groups of operators together, the mean microleakage scores for the three tested adhesives showed no statistical difference (*P* = .75). These findings were also valid for the MEO group (*P* = .25), but significant differences were found between the three tested adhesive systems in the EO and IO groups (*P* = .015 and *P* = .032, resp.). In the EO group, the mean microleakage score was lower for SB1 than for SBMP (*P* = .016) and ADSE (*P* = .0076), while in the IO group, the mean microleakage score was significantly higher for SB1 than for SBMP (*P* = .020) and ADSE (*P* = .027).

Table 7 shows the mean microleakage scores for the three tested adhesives at both interfaces (enamel and dentin). Considering the enamel and dentin interfaces separately, the mean microleakage scores for SBMP and SB1 were significantly lower at the enamel interfaces than at the dentin interfaces (*P* < .0001 and *P* < .0001, resp.). No difference was found between enamel and dentin for the ADSE adhesive system.

## 4. Discussion

*In vitro* studies are useful for predicting the clinical outcomes of adhesives [2, 13]. Such laboratory experiments enable comparison among different bonding materials to indicate statistical difference among different adhesive systems [3, 4, 14–26] and operators [5–11]. A previous study suggested that adhesive technique appears to be more operator dependant than technique sensitive [7].

The adhesive systems tested in the present *in vitro* study (SBMP, SB1, and ADSE) have been tested previously and found to be efficient in terms of mechanical properties and/or microleakage [3, 4, 15, 16, 19, 20, 22–24, 27–36]. In the present study, the mean microleakage scores were low (≤2), suggesting that microleakage of the adhesive restorations was clinically acceptable in all groups of operators and for all the tested adhesives.

In addition, the present data showed no significant difference between the periods before and after the operators being given the manufacturers' instructions for use of the adhesive systems. When all of the operators were considered together, there was no significant difference among the three tested adhesive systems in Part 1 of the study (before receiving instructions for use) or in Part 2 (after instructions for use) (Tables 5 and 6). Nevertheless, in the present study and according to the findings of Degrange and Lapostolle [7], significant differences were observed among the three groups of operators. In our study, when the three tested adhesives were considered together, mean microleakage score showed a statistically significant difference among the three different groups of operators: the mean microleakage score was significantly lower in the EO group than in the MEO (*P* = .0029) and IO groups (*P* < .0001); the mean microleakage score was lower in the MEO group than in the IO group (*P* = .023). This result could be explained by the low level of clinical experience (approximately 6 months) in the IO group at the time the study was conducted. Our results are consistent with those of other studies [8, 9, 11, 37–39] and suggest that clinical experience in the manipulation of adhesive systems could play an important role in the performance of adhesives.

In the first part of the present study, each operator performed adhesive restorations without any information regarding the adhesive or manufacturer's instructions other than his own knowledge and clinical experience. Before the beginning of the adhesive procedure, two supervisors (Sabine Geerts and Audrey Guéders) provided brief information concerning the name of the adhesive system (SBMP, SB1, or ADSE), the type of adhesion strategy (ER or SE), and the number of clinical steps required to apply the adhesive (2 or 3). The operators were asked to remain silent during the experiment. The results from this first part of the study showed very low mean microleakage scores for each adhesive in the EO group. In the MEO group, the mean microleakage score was significantly higher for SBMP than for the other two adhesives. Despite the fact that these students had not previously experimented with 3-step ER or with 2-step SE adhesive systems, their mean microleakage score was better for ADSE (a 2-step SE adhesive) than for SBMP. This result may indicate that SE adhesive systems are less technique-sensitive than are ER adhesives, as suggested previously [9, 10, 39, 40]. The students in the MEO group had already used a 2-step ER adhesive system in their clinical practice; consequently, the mean microleakage score reported for SB1 was not surprising in this group of operators. The mean microleakage scores were highest in the IO group, which could be related to the short period of clinical practice completed by these operators (approximately 6 months between beginning their clinical practice and taking part in the present study). In addition, for this least experienced group, in the first part of the study, there was no significant difference in mean microleakage score among the different adhesives. This result could be explained by the fact that these students had recently attended several courses and lectures concerning the principles of adhesion and the different commercially available adhesive systems.

In the second part of the present study, rigorous instructions on the use of the different tested adhesive systems were delivered to the participants before they were asked to perform the three adhesive restorations on the lingual side of each tooth. Our results showed no statistical difference in mean microleakage score between before and after delivery of the instructions, which is in disagreement with the findings of Finger and Balkenhol [41]. Therefore, following the manufacturer's instructions in using adhesive systems is very important for the success of adhesive restorations, but it is not the only important factor: other parameters include the distance between the tip of the air syringe and the surface of the cavity, orientation of the tip, and the air pressure of the syringe. Air pressure has a strong influence on the degree of air-drying of the dentin (which is an important step in the ER adhesion strategy), as does adequate evaporation of the solvents (which are present in all adhesive systems) [10]. Therefore, significant differences between operators may reflect the type of solvent used [9].

It appears that the present results are consistent with previous observations regarding the influence of the operator on the success of adhesive restorations [6, 7, 9, 39]. 

Regarding the interfaces of the restorations, the mean microleakage scores were significantly lower on the enamel interface than on the dentin interface for SBMP and SB1. This finding is not surprising because both of these adhesives are ER systems, which perform better on enamel tissue than do SE adhesive systems [6, 7, 13, 14, 19, 28]. On the dentin interface, the mean microleakage score was lower for ADSE than for SBMP and SB1. ADSE is an intermediary strong SE adhesives (pH = 1.5), and this type of adhesive generally shows good results on the dentin interface, as reported previously [1, 2]. 

Under the conditions tested, and based on the results obtained, it may thus be concluded that adhesive systems are operator sensitive but the influence of the operator varies with the adhesive system used, as reported previously by other authors [6, 7, 9, 39]. So, the present result that the lowest mean microleakage scores were found for the group of experts (EO) indicates the importance of clinical experience, which appears to have a greater influence on the quality of adhesive restoration than does the delivery of theoretical information containing the manufacturer's instructions. In addition, in the present experiment, the two tested ER adhesive systems (Scotchbond Multi-Purpose Plus and Adper Scotchbond 1 XT) showed less microleakage than the tested SE adhesive (AdheSE), but only on the enamel interface. Finally, the results of the present study indicate that the tested SE adhesive (AdheSE) gives good results in terms of microleakage and that within the limits of the study, this adhesive system appears to be less operator sensitive than are the other tested adhesives.

## Figures and Tables

**Table 1:** Characteristics of the tested adhesives.

Groups	Adhesives	Strategy of adhesion	Instructions for use
1 a	Scotchbond Multipurpose Plus (SBMP)	3-step ER	No
1 b	Scotchbond Multipurpose Plus (SBMP)	3-step ER	Yes
2 a	Adper Scotchbond 1 XT (SB1)	2-step ER	No
2 b	Adper Scotchbond 1 XT (SB1)	2-step ER	Yes
3 a	AdheSE (ADSE)	2-step SE	No
3 b	AdheSE (ADSE)	2-step SE	Yes

ER: Etch and Rinse adhesive system; SE: Self-Etch adhesive system.

**Table 2:** Sample identification: grooves drilled on the buccal surfaces of teeth.

Adhesives	Part 1 of the study	Part 2 of the study
SBMP	1 groove	No groove
SB1	2 grooves	No groove
ADSE	3 grooves	No groove

SBMP: Scotchbond Multipurpose: SB1: Adper Scotchbond 1 XT; ADSE: AdheSE.

**Table 3:** Mean microleakage scores according to operator skill for all adhesives, without taking into account the operator's level of knowledge of the adhesive system.

Groups of operators	n*	Mean microleakage scores (±SD)
EO	16	0.47 ± 0.59
MEO	11	0.58 ± 0.65
IO	14	0.67 ± 0.66

*P*		<.0001**

*Number of operators.

**Results statistically significant at *P* < .05.

EO: expert operators; MEO: moderate experience operators; IO: inexperienced operator.

**Table 4:** Statistical comparison between operator groups, without taking into account the operator's level of knowledge of the adhesive system.

	EO group	MEO group	IO group
EO group	—	—	—
MEO group	0.0029*	—	—
IO group	<0.0001*	0.023*	—

*Results statistically significant at *P* < .05.

EO: expert operators; MEO: moderate experience operators; IO: inexperienced operators.

**Table 5:** Mean microleakage scores according to operator skill for the three tested adhesives before instruction in their use.

Adhesives	Mean microleakage scores among the 3 operators groups			
	EO	MEO	IO	All
SBMP	0.48 ± 0.55	0.78 ± 0.75	0.62 ± 0.67	0.61 ± 0.66
SB1	0.45 ± 0.57	0.48 ± 0.52	0.78 ± 0.72	0.57 ± 0.63
ADSE	0.50 ± 0.58	0.43 ± 0.52	0.63 ± 0.64	0.53 ± 0.59

*P*	.68*	.0019*	.19*	.42*

*Results statistically significant at *P* < .05.

SBMP: Scotchbond Multipurpose; SB1: Adper Scotchbond 1 XT; ADSE: AdheSE.

EO: expert operators; MEO: moderate experience operators; IO: inexperienced operators.

**Table 6:** Mean microleakage scores according to operator skill for the three tested adhesives after instruction in their use.

Adhesives	Mean microleakage scores among the 3 operators groups			
	EO	MEO	IO	All
SBMP	0.54 ± 0.67	0.51 ± 0.61	0.58 ± 0.59	0.55 ± 0.63
SB1	0.32 ± 0.47	0.70 ± 0.75	0.79 ± 0.69	0.59 ± 0.66
ADSE	0.56 ± 0.68	0.59 ± 0.64	0.60 ± 0.62	0.58 ± 0.65

*P*	.015*	.25*	.032*	.75*

*Results statistically significant at *P* < .05.

SBMP: Scotchbond Multipurpose; SB1: Adper Scotchbond 1 XT; ADSE: AdheSE.

EO: expert operators; MEO: moderate experience operators; IO: inexperienced operators.

**Table 7:** Mean microleakage scores for the different adhesive systems at enamel and dentin interfaces.

Interfaces	Mean microleakage scores		
	SBMP	SB1	ADSE
Enamel	0.45 ± 0.66	0.46 ± 0.065	0.59 ± 0.64
Dentin	0.71 ± 0.60	0.70 ± 0.62	0.52 ± 0.60

*P*	<.0001*	<.0001*	.14*

*Results statistically significant at *P* < .05.

SBMP: Scotchbond Multipurpose; SB1: Adper Scotchbond 1 XT; ADSE: AdheSE.
